# Sustainable Health and Wellbeing in the European Union

**DOI:** 10.3389/fpubh.2022.851061

**Published:** 2022-03-16

**Authors:** Beata Gavurova, Silvia Megyesiova

**Affiliations:** ^1^Faculty of Mining, Ecology, Process Control and Geotechnologies, Institute of Earth Resources, Technical University of Košice, Košice, Slovakia; ^2^Faculty of Business Economy, University of Economics, Bratislava, Slovakia

**Keywords:** public health, wellbeing, sustainable development, European Union, TOPSIS method, ranking method

## Abstract

**Background:**

Altogether, 17 Sustainable Development Goals (SDGs) are an urgent call for action to end poverty, protect the planet, and ensure prosperity for all. Goal 3 is crucial in terms of good health and wellbeing. The main aim of this study is to analyze and evaluate differences among indicators of SDG 3: Sustainable health and wellbeing in the EU countries.

**Methods:**

The status and development of the EU Member States regarding their successes or failures in terms of Goal 3 were subjected to analysis. Altogether, 11 indicators were used to rank the EU countries using the TOPSIS and ranking methods. The ranks were assigned to the countries in two periods. The first period is related to the time from 2010 till 2014, and the second period from 2015 till 2019.

**Results:**

The EU countries achieved a positive development in 10 of 11 indicators that monitor the achievement of the EU in terms of Goal 3. The only variable that changed negatively was the obesity rate. Positivity was observed in the decline of the standardized preventable and treatable mortality, which declined from 317.3 in the first period to 295 in the second period; the drop of the population weighted annual mean concentration of fine particulate PM2.5, from 16.4 to 13.6 μg/m^3^, and also in the increase of the share of people with good or very good perceived health, which was combined with a decrease of the self-reported unmet need for medical examination and care. The best-rated country in terms of SDG 3 was, in both periods, Sweden, while the worst-rated was Latvia.

**Conclusions:**

Governments and institutions in the EU can intervene to increase the accessibility and quality of the health care system, but every citizen should try to do their best to reduce some of the risk factors, such as smoking or obesity, to try living healthier and to help to achieve higher ambitions in terms of sustainable health and wellbeing.

## Introduction

Sustainability of health and wellbeing of population represents one of the main aims of sustainable development. The COVID-19 pandemic has significantly impacted countries, destabilized global economy, and threatened lives of billions of people all over the world. This crisis revealed the countries' readiness to solve health emergencies and to invest in critical public services. Many research studies and reports advised health improvement of the population on a global scale before the COVID-19 pandemic. The indicators—an increase in life expectancy, reduction in child and maternal mortality, etc.—show evident improvement of health. Lifestyle changes, rise of civilization, and chronic diseases require creation and monitoring of health policies that would improve quality and availability of health care. Availability of health care is also related to financial availability and sustainability in health systems.

Measuring and quantifying the level of health and wellbeing within countries is a long-term subject of research of many research teams, economists, and international institutions (1–6). Many authors point to procedural and methodological difficulties of these measurements (7–9). Similarly, knowledge of countries' health systems and their determinants is a very important aspect in this process. It is also a result of the fact that a health system of a particular country is strongly influenced by its level of technological progress, economic and social aspects, political situation, etc. Sustainable health system has to be defined by at least three key indicators: availability to each individual, mutual acceptance between patients and health care personnel, and socio-economic and demographic changes in the country. Innovative development, which will enable improvement of quality and effectiveness of health care services, has a significant impact on sustainability of health systems (10–12). Brӑtucu et al. (13) link innovative development with the Sustainable Development Goals (SDGs) and with Agenda 2030 in their study. As these authors (13) suggest, there exists a direct relationship between a way of how people evaluate their personal health and an ability to browse Internet in order to search for information about health. Authors appeal to industrial specialists, academics, and public authorities in creating sustainable health systems and policies that would enable a development of adequate mobile health care programs and take better care of one's health. Consequently, increasing sophistication of new technologies changes availability and management of health services and information. Additionally, it will limit an ability of a company to offer equitable access to health services for all (13–15). In recent years, issue of sustainable health has been researched in relation to efficiency of health systems [e.g., (16, 17)] and also in relation to quality of life (18, 19). However, as the results of research studies between countries show, there exist significant differences. For instance, research results by Rogge and Nijverseel (19) show a clear divide between the Nordic and Western European countries and the Southern and Eastern European countries, with people in the former countries experiencing quality of life to be higher as compared to people in the latter countries.

The health systems' goal in the individual countries is to provide a sustainable health system that would enable access to high-quality health care, equity, and solidarity for the entire population. Fiscal sustainability represents one of the dimensions that evaluates the sustainability of health systems in the countries. Fiscal sustainability of health systems will be a significant problem in the future, according to many experts and research results. It is related to processes of global demographic aging, increasing investments into technologies and infrastructure, innovative medical products, etc. Thus, this issue is frequently connected to macroeconomic parameters, which is also confirmed by studies that examine a relation between economic growth and health, and also degrowth and health (20–23).

The consequent facts indicate a strong significance of the examined topic, sustainable health, and wellbeing, while its importance increases during the pandemic crisis. The above-mentioned facts represent a motivation of authors to realize this research, which aims at analyzing and evaluating the differences in 11 indicators of SDG 3: sustainable health and wellbeing in the EU countries during two monitored periods. The results of this study are beneficial for creators of health and economic policies, and also for creators of national and strategic plans. Similarly, the study results will support a creation of national and international benchmarking indicators and result in a realization of comparative analyses. Consequently, this will enable a creation and development of multidisciplinary research teams, which would focus on a development of methodologies in this area, and a formation of particular databases.

## Materials and Methods

Sustainable development is one of the key issues of the world and the European Union (24, 25), with an effort to shape a more sustainable, better, and safer world for all (26, 27). The success in achieving goals of sustainable development is in the interest of the EU, of the Member States, of the EU regions, and naturally, of the whole European population. To measure the state, the development, and the direction of sustainable development, a set of indicators was chosen by the EU authorities (24–26, 28, 29). A group of indicators was selected for each of the 17 SDGs. In the study, the focus was targeted on the development and state of SDG 3: good health and wellbeing. The analysis of the EU Member States regarding the set of indicators was realized in two periods. The first period represents the average values of indicators for the time span from 2010 until 2014 (i.e., average of the indicators for five consecutive years), and the second period represents the average values for the time span between 2015 and 2019 (average value of variables for 5 years). In case that some of the indicators were not published chronologically for the time span between 2010 and 2014 or 2015 and 2019, the average value of an indicator was calculated according to availability of the dataset. The dataset of SDG 3 indicators used for analysis of the status of 28 EU countries was downloaded from the Eurostat database (30). The longer time span allowed to follow the changes of the selected variables in the EU and the successes or failures in achieving the goals of sustainable development. The EU consists of 27 countries nowadays, but altogether, 28 countries were subjected to analysis due to the fact that the United Kingdom left the EU on 31st January 2020, and during the analyzed period of time, the UK was still a member of the EU. The countries were ranged from the best to the worst using two appropriate methods suitable to determine the ranking based on a multidimensional view of the achievement of SGD 3. The Technique for Order Preference by Similarity to an Ideal Solution (TOPSIS) and the ranking methods were used for the normalization of the analyzed indicators. The ranking method belongs to the simplest normalization approach; it is the simplest evaluation method and is based on rank of each indicator across countries (31). The lowest rank, the best rank, is assigned to a country with the most optimal level of the indicator values, while the highest rank is assigned to a country with the worst level of the indicator values. After each selected indicator across countries is ranked, an overall average of the assigned rank values is calculated for the analyzed countries. The advantage of this method is its simplicity and the fact that it is independent to outliers (31–33). The disadvantages of ranking method are the loss of information of the absolute values of the indicators and the impossibility to evaluate any outcome about difference in performance and/or about gap in indicator levels among countries. The next used multi-criteria decision method was TOPSIS. This method is more demanding compared to the ranking method but enables to measure the relative performance for each alternative, to rank the alternatives based on the shortest distance from the positive ideal solution and the farthest from the negative ideal alternative (33, 34). The decision matrix (35, 36) in the study is contracted with 28 alternatives: it means 28 EU countries and 11 criteria. The weight of criteria and the weight of indicators were set as equal. The next step of the analysis was dedicated to the calculation of the normalized decision matrix, identification of positive and negative ideal solutions, followed by calculation of distances of each alternative from the ideal positive and ideal negative solutions (37–39). In the last step, the synthetic measure of relative closeness for individual alternatives was defined. The relative closeness can be equal or higher than 0 and equal or lower than 1 (34–40). The alternative with the synthetic measure closest to 1 represents the best object, the best country. Using the synthetic measure, the alternatives, in our case the group of EU countries, can be ranked by the descending order. The detailed steps of the TOPSIS method with equal weight of the criterions are listed below (33–40):

Step 1: Establish the criteria matrix *X* = (*x*_*ij*_)


$$\begin{array}{l} X=\left[\begin{array}{cccc} x_{11} & x_{12} & \ldots & x_{1n}\\ x_{21} & x_{22} & \ldots & x_{2n}\\ \vdots & \vdots & \vdots & \vdots \\ x_{m1} & x_{m2} & \ldots & x_{mn} \end{array}\right];\text{i}=1,2,\ldots ,\text{m};\text{j}=1,2,\ldots ,\;\text{n,} \end{array}$$

where *x*_*ij*_ is the value of alternative, of object, with respect to criterion evaluated.

Step 2: Calculate the normalized decision matrix *R* = (*r*_*ij*_)


$$\begin{array}{l} r_{ij}=\frac{x_{ij}}{\sqrt{\overset{m}{\underset{i=1}{\sum }}x_{ij}^{2}}} \end{array}$$

Step 3: Identify the positive ideal solution *A*^+^ and the negative ideal solution *A*^−^


$$\begin{array}{l} A^{+}=\left\{r_{1}^{+},r_{2}^{+},...,r_{n}^{+}\;\right\}=\left\{\left(\max r_{ij}|\;j\in I\right),\left(\min r_{ij}|\;j\in J\right)\right\}\\ A^{-}=\left\{r_{1}^{-},r_{2}^{-},...,r_{n}^{-}\;\right\}=\left\{\left(min\;r_{ij}|\;j\in I\right),\left(max\;r_{ij}|\;j\in J\right)\right\},\; \end{array}$$

where *I* is associated with benefit, stimulant criteria, and *J* is associated with cost, destimulant criteria; *i* = 1, 2, …, *m*; *j* = 1, 2, …, *n*.

Step 4: Calculate the separation measures according to the Euclidean distance of each alternative from the positive ideal and the negative ideal solutions


$$\begin{array}{l} d_{i}^{+}=\;\sqrt{\overset{n}{\underset{j=1}{\sum }}{\left(r_{ij}-r_{j}^{+}\right)}^{2}};\text{i}=1,2,\ldots ,\;\text{m}\\ d_{i}^{-}=\;\sqrt{\overset{n}{\underset{j=1}{\sum }}{\left(r_{ij}-r_{j}^{-}\right)}^{2}};\text{i}=1,2,\ldots ,\;\text{m} \end{array}$$

Step 5: Determinate the synthetic measure of relative closeness to the ideal solution


$$\begin{array}{l} C_{i}=\frac{d_{i}^{-}}{d_{i}^{-}+d_{i}^{+}},\; \end{array}$$

where *i* = 1, 2, …, *m* and 0 ≤ C_*i*_ ≤ 1.

Step 6: Finally, a set of alternatives can be ranked by the descending order of the *C*_*i*_ values. The highest value of the *C*_*i*_ indicates the best alternative, while the lowest value of *C*_*i*_ indicates the worst object.

The association of ranks of the EU countries that were assigned to the countries by two methods was measured using the Spearman's rank-order correlation coefficient. It is a suitable technique to identify the association between two ranked ordinal variables (41, 42). The Spearman's correlation coefficient can take a range of values from −1, in case of perfect negative correlation, to +1, in case of perfect positive correlation between ranks (41–43). The used methods enable to rank the EU countries according to the selected criteria and to follow the competitiveness of the countries (44, 45).

## Results

The state and performance of SDG 3—Good health and wellbeing in the EU is measured using a set of eleven indicators (24–26). These indicators were downloaded from the Eurostat database from 2010 till 2019 (30). The variables are either stimulants or destimulants. From the total collection of indicators, two are stimulants and nine are destimulants. An indicator is stimulant if its maximization is considered as a positive state, while an indicator is destimulant if its minimalization is considered as a positive condition. The following eleven indicators of the SDG 3 were included in the study (24–26):

x_1_ healthy life years (HLY) in absolute value at birth,

x_2_ share of people with good or very good perceived health,

x_3_ smoking prevalence,

x_4_ standardized death rate due to tuberculosis, HIV and hepatitis,

x_5_ standardized preventable and treatable mortality,

x_6_ self-reported unmet need for medical examination and care,

x_7_ obesity rate by body mass index (BMI),

x_8_ people killed in accidents at work,

x_9_ population living in households considering that they suffer from noise,

x_10_ road traffic deaths,

x_11_ exposure to air pollution by particulate matter.

The first two indicators belong to stimulants, while the other indicators belong to destimulants. The indicator x_1_ measures the number of remaining years that a person of specific age is expected to live without any severe or moderate health problems, and it is also called a disability-free life expectancy indicator. HLY at birth is stimulant, and the increase of the HLY is one of the main goals for the European health policy. The indicator x_2_ represents the share of population aged 16 or over, perceiving itself to be in very good or good health; the data are collected from the EU Statistics on Income and Living Conditions (EU SILC). For variables x_1_ and x_2_, it is positive to have the highest possible values. The first two indicators can monitor and predict the future health care needs, care demand, and future mortality. The next briefly described indicators are destimulants. The indicator x_3_ is calculated as the proportion of the population aged 15 years and over, who report that they currently smoke tobacco products. These data do not include use of the electronic cigarettes and snuff. Indicator x_3_ is collected through a Eurobarometer survey; therefore, the data availability is limited. The published dataset of indicator x_3_ consists of the following years: 2012, 2014, 2017, and 2020. Thus, the average value of the indicator of the first period is calculated as an average value for years 2012 and 2014, and the mean value of the second period is calculated as the average value for years 2017 and 2020. The indicators x_4_ and x_5_ characterize the standardized death rates (SDRs) for selected diseases per 100,000 inhabitants. The SDRs are the rates adjusted to the European population; their comparability can be improved over time and among countries. The variable x_4_ represents the SDR of tuberculosis, HIV, and hepatitis, and is calculated by dividing the number of people dying due to selected diseases by the total population. The indicator x_5_ measures the SDR of preventable and treatable diseases. Treatable and preventable mortality belong to avoidable mortality. Preventable mortality could be avoided through primary prevention interventions and effective public health system, and the treatable mortality could be avoided through early and highly effective health care intervention and treatment. The aim of the EU countries is to minimize both mortality rates; it means minimizing the indicators x_4_ and x_5_. The variable x_6_ is the ratio of the population aged 16 and over, which, due to financial reason, due to waiting list, or due to the territorial distance, reported unmet needs for medical care. Obesity rate belongs to risk factors of health; therefore the indicator x_7_ belongs to the indicators of the SDG 3. The obesity rate is the proportion of persons aged 18 and over that had the BMI ≥ 30. The Period 1 for the indicator x_7_ consists of years 2008 and 2014, while the Period 2 consists of years 2017 and 2019. The reason of the accessibility of the obesity rate being limited only to a few periods is the fact that this information is collected by European Health Interview Survey (EHIS), and the harmonized survey is conducted only in waves with a specific time spacing (46). The indicator x_8_ measures the number of fatal accidents that occur during work per 100,000 persons in employment. The ratio of the population who declare that they are affected by noise from the street or affected by the noise from neighbors represents the variable x_9_. The indicator x_10_ is measured by the number of fatalities caused by road accidents per 100,000 persons (number of deaths up to 30 days after the occurrence of the road accident). From the environmental and public health view, the air pollution is an important factor that significantly affects the health status of the population. Therefore, the last indicator, x_11_, measures the population weighted annual mean concentration of fine particulates PM_2.5_ at urban background stations in agglomerations. The exposure to PM_2.5_ causes more serious health deterioration than exposure to PM_10_, as the very small particulates with the diameters <2.5 μm are more toxic and can be carried deeper into the lungs, therefore causing serious lung and heart disease. The aim of the EU countries is to maximize the values of the indicators x_1_ and x_2_, and, on the other hand, to minimize the values of indicators x_3_-x_11_.

### Status and Development of the SDG 3 Indicators

The summary statistics of the indicators in the first period are presented in Table 1.

**Table 1:** Summary statistics of indicators in period 1 (2010-2014).

**Variable**	**Mean**	**Std dev**	**Minimum**	**Maximum**	**Range**
x_1__period 1	61.5	4.2	53.4	71.7	18.2
x_2__period 1	65.9	10.3	46.1	82.8	36.8
x_3__period 1	27.1	5.2	12.0	39.0	27.0
x_4__period 1	3.3	2.7	0.9	10.0	9.1
x_5__period 1	317.3	127.9	187.3	584.4	397.1
x_6__period 1	3.7	3.6	0.1	14.0	13.9
x_7__period 1	16.4	3.2	8.7	24.5	15.8
x_8__period 1	2.5	1.2	0.7	5.6	5.0
x_9__period 1	17.7	5.4	9.2	29.8	20.5
x_10__period 1	6.3	2.3	2.9	10.1	7.1
x_11__period 1	16.4	5.8	6.8	30.8	24.1

The HLY at birth reached on average 61.5 years; the best country with the highest HLY was Malta, and the worst was Slovakia. The share of people with good or very good perceived health was as high as 65.9% in the EU, with the highest proportion in Ireland and the lowest in Lithuania. The first two mentioned variables were stimulants; therefore, the best country was the country with the highest maximal, value of the indicator. The next indicators are destimulants; it means that the best country is the country with the minimal value of the analyzed variable. The smoking prevalence was the lowest in Sweden, the highest one in Greece, and the overall average for the EU countries was 27.1%. The SDRs of indicators x_4_ and x_5_ are very low in the “older” EU member states and high in the “new” EU countries. On average the indicator x_4_ reached 3.3, but in Latvia, it was 10.0, and in the Netherlands, only 0.9. Very large reserves had the post-communist countries in the field of preventable and treatable mortalities. The standardized preventable and treatable mortality was in Hungary, Romania, Lithuania, and Latvia, which was higher than 530, but on the other hand, it was lower than 200 in Spain, Italy, and Cyprus. So, the differences between the EU countries were in terms of the indicator x_5_ being very high. The ratio of the self-reported unmet need for medical examination and care was acceptably low in Slovenia, Austria, the Netherlands, but unfortunately, it was very high in Romania (11.1%) and Latvia (14.0%). Obesity is a risk factor for health outcome and so the obesity rate is in interest of the SDG 3. The average EU obesity rate reached 16.4%, with the minimal value in Romania (only 8.7 %) and the highest, worst, share in Malta, which stood at 24.5%. The fatal accidents at work averaged at 2.5 per 100,000 persons in employment. In the most developed EU countries, the rate was very low, but, for example, in Lithuania, it stood at 4.5, and in Romania, at 5.6. The proportion of EU population living in households considering that they suffer from noise was, in the first period, as high as 17.7%, but the worst situation was measured in Malta (29.8%). The road traffic deaths were low in the United Kingdom and Sweden, where the indicator was 2.9 per 100,000 persons, but it was as high as 9.6 in Poland and even higher in Romana (10.1). Some EU countries, especially the post-communist countries, unfortunately reached high levels of the road traffic deaths. The exposure to air pollution has negative consequences on population health; therefore, it is in the interest of the countries to minimize air pollution. The indicator x_11_ measures the concentration of the fine particulates PM_2.5_, whose diameters are <2.5 μm and are very dangerous for human being. Very good results in relation to this indicator were achieved in Sweden with the exposure at 6.8 μg/m^3^ and Finland (7.6 μg/m^3^). On the other hand, the worst rated countries had an extremely high levels of exposure to fine particulates; in Poland, it was 27.3 μg/m^3^, and in Bulgaria, 30.8 μg/m^3^.

The summary statistics of the eleven indicators in the second period are presented in Table 2.

**Table 2:** Summary statistics of indicators in period 2 (2015-2019).

**Variable**	**Mean**	**Std dev**	**Minimum**	**Maximum**	**Range**
x_1__period 2	61.9	5.0	52.7	72.9	20.2
x_2__period 2	66.7	9.8	44.1	83.4	39.3
x_3__period 2	24.9	7.2	7.0	39.5	32.5
x_4__period 2	2.7	2.5	0.6	10.6	10.0
x_5__period 2	295.0	118.3	176.4	532.5	356.1
x_6__period 2	2.9	3.2	0.2	14.3	14.2
x_7__period 2	17.7	3.6	10.7	27.2	16.6
x_8__period 2	2.1	1.0	0.5	4.4	3.8
x_9__period 2	16.1	5.1	8.3	26.4	18.1
x_10__period 2	5.5	1.9	2.6	9.7	7.1
x_11__period 2	13.6	4.6	5.6	23.0	17.3

The HLY at birth reached on average 61.9 years; the best country with the highest HLY was Sweden, and the worst was Latvia. The indicator x_2_ was as high as 66.7% in the EU, and the position of the best and the worst countries was the same as in the first period; hence, the best EU member was Ireland, and the worst was Lithuania. The average ratio of the smoking prevalence reached 24.9%, which is a positive decline when compared with the value in the first period; the positions of the best and worst countries according to the indicator x_3_ did not change. The average value of the indicator x_4_ dropped to 2.7, but still was the range between the best country, the Netherlands, and the worst country, Latvia. In the case of the variable x_5_, the differences between the countries are extremely high; for example, in Italy, the standardized preventable and treatable mortality was only 176.4, while in Latvia, it was as high as 532.5. The unmet need for medical examination and care was also very high in the second period; for example, it reached 10.5% in Greece and 14.3% in Estonia. The best rated countries, the Netherlands and Austria, on the other hand, had the indicator x_6_ at only 0.2%. The average of the indicator x_7_ increased to 17.7%; the ratio for the best rated country, namely Romania, increased from 8.7% in the first period to 10.7% in the second period. The worst country in terms of obesity, Malta, showed a deterioration too, since the obesity rate increase from 24.5 to 27.2%. Obesity rising has become a common public health crisis among the developed countries. The indicator x_8_ ranged from 0.5 (Netherlands) till 4.4 (Romania), with an average value of the people killed in accidents at work at 2.1 per 100,000 persons in employment. The variable x_9_ reached an average value of 16.1%; its lowest level in Croatia was only 8.3%, and the highest in Malta stood at 26.4%. Also, in the second period, the post-communist countries were faced with a high ratio of the road traffic deaths since it was as high as 7.7 per 100,000 persons in Poland and Croatia, 7.8 in Latvia, 9.4 in Bulgaria, and the worst situation was in Romania, with the maximum of 9.7. Whether the high death rates are associated with a poorer quality of roads, a lack of motorway networks, worser quality of vehicles used, or riskier drivers' behaviors, all these factors should be in the interest of these countries, with the aim to minimize higher road traffic deaths rates. The exposure to air pollution by fine particulate matter averaged in the EU at 13.6 μg/m^3^. In the best country, in Finland, it was only 5.6 μg/m^3^, but in Poland, it was as high as 23 μg/m^3^.

In Table 3, the relative and absolute changes of the indicators are presented. Positive development of a stimulant indicator is its growth, while a positive development of a destimulant variable is its decline. According to the relative changes, the indicator x_1_ grew by 0.7% (by 0.4 years) between the two analyzed periods. The increase is not very high, but the important thing is that it changed in a positive way. The share of European population that is satisfied with their health increased by 1.2%; it means by 0.8% points. It can be said that both stimulants changed in a positive way, as both variables increased from the first to the second period. The smoking prevalence is a very important indicator; it decreased by 8.1%, and it will definitely have a positive impact on population health in the future. The EU countries were very successful in declining both indicators related to death rates. The SDRs due to tuberculosis, HIV, and hepatitis dropped by 18.2%, and the preventable and treatable mortality shrank by 7%. A strong relative decline was measured in case of the indicator x_6_; it means that the European population had a better access to medical examination and care in the second period compared with the first period. The decline of the mortality rates (indicators x_4_ and x_5_) could be related to a better and easier medical availability, as this is the result of the change in indicator x_6._ The mortality decline can be related also to the decline of smoking prevalence and other unmeasured variables. All the mentioned factors could also result in an increase of the population that is in good or very good health (indicator x_2_). The obesity rate is the only one indicator that changed in a negative way. It is a destimulant variable, and so in case of a favorable development, we would expect a drop of its value. However, an opposite situation was observed in the EU countries. The variable x_7_ increased by 7.9% (by 1.3% points); thus, the obesity crisis is still a problem for the health care system of the European countries. Unfortunately, obesity negatively affects a person's health and will result in morbidity and can lead to mortality of a human being. The next variable x_8_ declined, and it is a good sign for the sustainable development in terms of the SDG 3. The decline of the ratio of people killed in accidents at work shows a positive move to a better and safer quality of the working environment in the EU. Additionally, the share of population living in household considering that they suffer from noise declined. The indicator x_10_ changed positively and the decline of the road traffic death by 12.7% is a success for the analyzed countries. From the quality of life, environment and health perspectives are rated very positively, as the exposure to air pollution by fine particulate matter dropped by 17.1%. Overall, it can be said that the development of the analyzed variables of the SDG 3 was positive, and only in one case (indicator x_7_), the change is rated negatively.

**Table 3:** Relative and absolute changes of the indicators between period 1 and period 2.

**Variable**	**Relative change, %**	**Absolute change**
x_1_	0.7	0.4
x_2_	1.2	0.8
x_3_	−8.1	−2.2
x_4_	−18.2	−0.6
x_5_	−7.0	−22.3
x_6_	−21.6	−0.8
x_7_	7.9	1.3
x_8_	−16.0	−0.4
x_9_	−9.0	−1.6
x_10_	−12.7	−0.8
x_11_	−17.1	−2.8

### Ranking Using the TOPSIS Method

The TOPSIS method was used to rank the EU countries from the best to the worst according to their achievements in field of Goal 3. Country rankings for both periods are shown in Table 4.

**Table 4:** Ranks of the EU countries by the TOPSIS method.

**TOPSIS**			
**Country**	**Period 1**	**Period 2**	**DIFF** **(period 2** – **period 1)**
SE	1	1	0
DK	2	2	0
NL	3	3	0
UK	4	5	1
FI	5	8	3
IE	6	4	−2
BE	7	6	−1
DE	8	10	2
LU	9	17	8
ES	10	7	−3
SI	11	12	1
FR	12	15	3
SK	13	14	1
CZ	14	13	−1
MT	15	11	−4
AT	16	16	0
CY	17	9	−8
HU	18	18	0
HR	19	20	1
EL	20	25	5
IT	21	19	−2
PT	22	22	0
PL	23	21	−2
EE	24	27	3
BG	25	23	−2
LT	26	24	−2
RO	27	26	−1
LV	28	28	0

*Country codes: BE, Belgium; BG, Bulgaria; CZ, Czech Republic; DK, Denmark; DE, Germany; EE, Estonia; IE, Ireland; EL, Greece; ES, Spain; FR, France; HR, Croatia; IT, Italy; CY, Cyprus; LV, Latvia; LT, Lithuania; LU, Luxembourg; HU, Hungary; MT, Malta; NL, the Netherlands; AT, Austria; PL, Poland; PT, Portugal; RO, Romania; SI, Slovenia; SK, Slovakia; FI, Finland; SE, Sweden; UK, the United Kingdom*.

**Figure 1:**
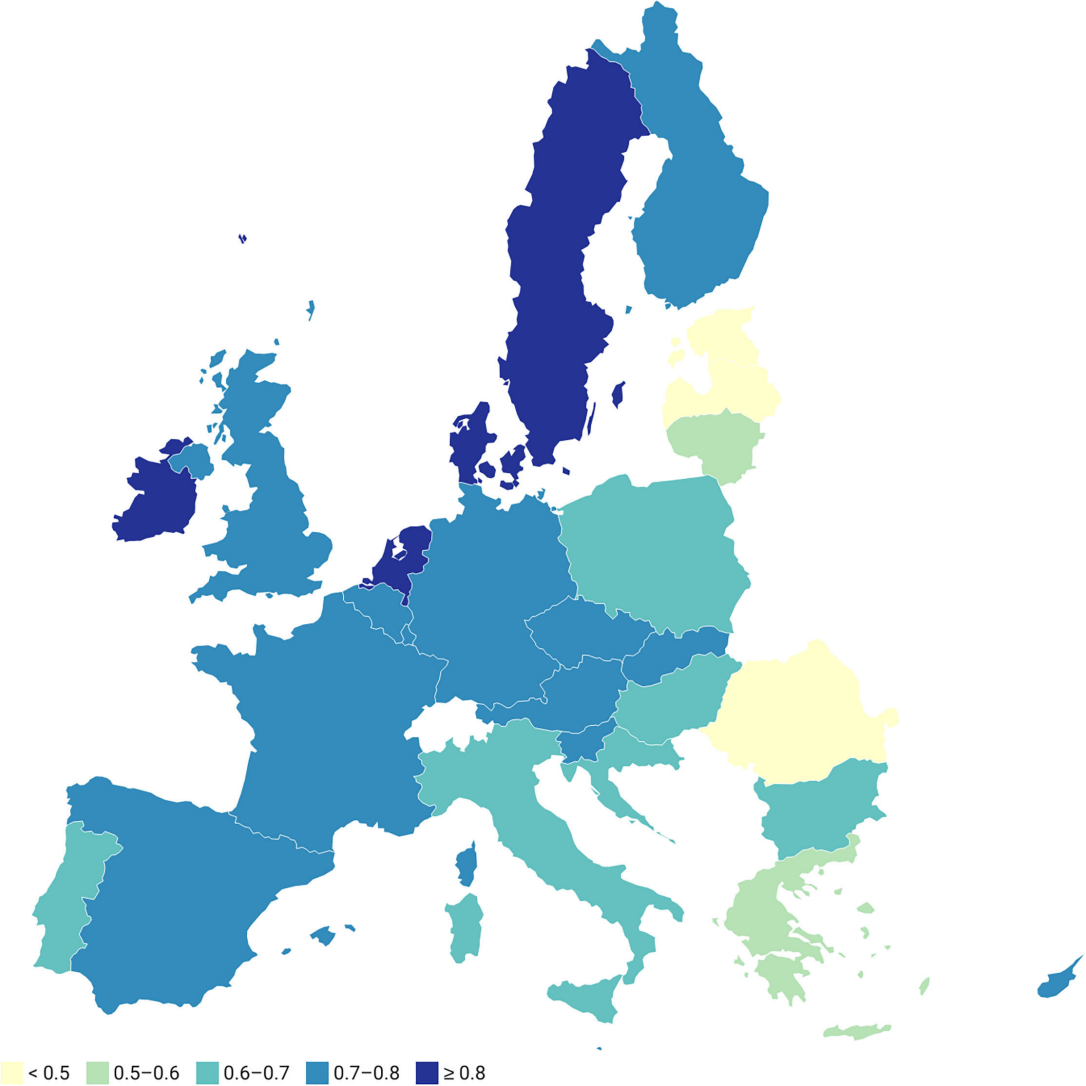
Relative closeness of the EU countries with respect to the ideal solution, 2nd period.

The best country is the one with the highest score of the relative closeness; it means the highest index C_i_, while the worst country is one with the lowest value of C_i_ (11–17). Among the best countries in the first period are Sweden, Denmark, and the Netherlands. These countries had the relative closeness C_i_ to the ideal solution higher than 0.8. The index C_i_ between 0.7 and 0.8 (ranks 4–12) points a very good position of the countries in terms of good health and wellbeing of the population. The countries with a score of C_i_ equal or higher than 0.6 and lower than 0.7 are countries with positions 13–18. The most of the new EU countries reached the C_i_ lower than 0.6; Greece, Italy, and Portugal were ranked among these countries. The worst situation in good health and wellbeing in the first period was achieved in Lithuania (C_i_ = 0.463), Romania (C_i_ = 0.291), and Latvia (C_i_ = 0.241); these three countries were ranked as the three worst countries among the EU Member States. In the second period, the ranking of the countries according to the indicators of Goal 3 was very similar compared to the first period. However, some countries improved their positions, while some worsened their positions. No change in ranking occurred in case of the first three best rated countries, namely Sweden, Denmark, and the Netherlands. These countries exhibited an exceptionally good situation regarding the indicators of Goal 3. An improvement or deterioration of positions not higher than four in case of a larger analyzed time span is not considered as significant. However, in case of Luxembourg, Cyprus, and Greece, the changes of their positions between the first and the second period are significant. Greece was ranked as 20th in first period but due to a deterioration of its indicators compared to the other EU countries; it ranked only 25th in the second period. The comparison of the TOPSIS ranks for Luxembourg was very surprising. In the first period, Luxembourg ranked 9th, but in the second period, its position changed to 17. The deterioration of Luxembourg's position was very high; however, the change of its index C_i_ was not so dramatic, as it declined from 0.731 to 0.705. The significant decrease of Luxembourg's position was due to an increase of the relative closeness C_i_ to the ideal solution of some other EU countries and due to the fact that the change of some indicators between the first and the second period was not in a positive sense. On the other hand, the position of Cyprus changed in a very positive way, from position 17 in the first period to position 9 in the second period. The positive movement was related to an increase of the index C_i_ from 0.646 to 0.766. In the second period, the three worst countries according to the situation in good health and wellbeing were Romania (C_i_ = 0.422), Estonia (C_i_ = 0.400), and Latvia (C_i_ = 0.352). Again, the new EU countries were very negatively ranked according to the results of the TOPSIS method (see Figure 1).

### Ranking Method

The second method used for ranking of the EU countries from the good health and wellbeing perspective was the ranking method. This method allowed to rank the countries, but it does not allow to follow any changes and values of a special index as was the case in the TOPSIS method. Sweden was the best rated country in the first period, not only using the TOPSIS method but also the ranking method (see Table 5). The rankings of other countries were very similar too. The first eight best positions in the first period belonged to the older EU Member States. This fact indicates that the situation in health and wellbeing is better rated in the older, more developed EU countries. Among the new EU countries, Malta got the best position, followed by Cyprus and Slovakia. From the older EU members, the worst countries were Greece and Portugal. Very similar ranks were assigned to the countries using the TOPSIS method. The three overall worst countries did not change when compared with the TOPSIS method. Again, the worst countries were Lithuania, Romania, and Latvia.

**Table 5:** Ranks of the EU countries by ranking method.

**Ranking method**			
**Country**	**Period 1**	**Period 2**	**DIFF** **(period 2** – **period 1)**
SE	1	1	0
NL	2	2	0
IE	3	3	0
DK	4	5	1
UK	5	7	2
ES	6	4	−2
FI	7	9	2
LU	8	14	6
MT	9	10	1
BE	10	8	−2
FR	11	13	2
CY	12	6	−6
SK	13	19	6
IT	14	11	−3
AT	15	16	1
DE	16	12	−4
EL	17	17	0
CZ	18	18	0
SI	19	15	−4
PT	20	21	1
EE	21	20	−1
BG	22	22	0
HU	23	23	0
HR	24	25	1
PL	25	24	−1
LT	26	26	0
RO	27	27	0
LV	28	28	0

The analysis in the second period did not bring any differences for the first three countries and for the last three countries. In the second period, the best rated countries were Sweden, the Netherlands, and Ireland, while to the worst rated countries were Lithuania, Romania, and Latvia. It means that also in the second period, the most developed, the old EU members, were more positively evaluated in terms of meeting the objectives of Goal 3. Between both periods, no changes in ranks for some countries were observed; in case of other countries, a moderate change was typical. Only in case of three EU countries, the change of ranks among two periods was significant, namely for Luxembourg, Cyprus, and Slovakia. Luxembourg and Cyprus changed their positions significantly also using the TOPSIS method. Luxembourg deteriorated its position from 8 in the first period to 14 in the second period, while Cyprus improved its position from 12 to 6 in the same time span. Slovakia experienced a deterioration from position 13 in the first period to position 19 in the second period. Among the 10 best rated countries, only Cyprus and Malta featured from the group of new EU Member States, and among the 10 worst rated countries, only Portugal found its location from the group of the older EU countries.

The Spearman's correlation coefficient was used to measure the strength of association between the ranks of the two methods used and between the two analyzed periods. The Spearman's correlation presented in Table 6 is positive and very high, and it is also statistically significant. The highest correlation between the ranks determined in the first and the second periods was discovered for the TOPSIS method (r = 0.935) and for the ranking method (r = 0.952). The correlation between the TOPSIS and ranking method in the first (r = 0.894) and in the second period (r = 0.902) was also very high. Therefore, the ranks were set very similarly in the first and in the second period, and they were set very similarly in case of the TOPSIS and the ranking methods.

**Table 6:** Spearman's correlation between the used ranking methods.

**Spearman Correlation Coefficients, N** **=** **28** **Prob** **>** **|r| under H0: Rho=0**				
	**TOPSIS period1**	**Ranking** **period 1**	**TOPSIS** **period 2**	**Ranking** **period 2**
TOPSIS_period1	1.000	0.894 <0.0001	0.935 <0.0001	0.854 <0.0001
Ranking_period 1	0.894 <0.0001	1.000	0.880 <0.0001	0.952 <0.0001
TOPSIS_period 2	0.935 <0.0001	0.880 <0.0001	1.000	0.902 <0.0001
Ranking_period 2	0.854 <0.0001	0.952 <0.0001	0.902 <0.0001	1.000

## Discussion and Conclusion

Good health and wellbeing are in the interest of every individual, governments, and institutions all over the world. Active health policy in each country is closely related to health sustainability of population. EU health policy has had a new macroeconomic perspective in recent years. It is related to analyses of budgets and financial obligations of governments in the individual countries and also to identification of policies and those factors that are critical for sustainability of health systems (47, 48). Macroeconomic dimension of health is also related to individuals, institutions, and health resources. In recent years, there have been active debates on optimal mechanisms of functioning and provision of universal access to high-quality health services (49–52). In the past, many countries have made major reforms in their health systems in order to improve availability and quality of health services for all (53, 54). However, sources of funding to date are insufficient and too costly. Consequently, countries have to identify the challenges of their health systems effectively and have to perform analyses of their sustainability. As a part of these analyses, it is very important to examine and to reveal the determinants that have a significant impact on sustainability of health systems, and, based on these findings, to set optimal health policies (50, 55, 56). Health systems are complex heterogenous systems, and their ability to use a combination of available resources and to transform them into results in health care area depends on many aspects, such as economic and social condition of a country, institutional system setting, development level of a country, political stability, population health status (mortality and morbidity structure), etc. (48, 57–59). Consequently, research of health systems' parameters and their indicators represents a fundamental platform for a creation of efficient policies that would lead to population health improvement and its sustainability (60, 61). Last but not the least, it would enable reducing international, national, and regional differences in health, which also represents one of the WHO's priorities (62). Additionally, comparative analyses of health systems, which enable identification of national differences and their causes, play a very important role (63).

The importance of these goals is intensified by the current pandemic situation caused by COVID-19 and strong pressures to create quality mechanisms in prevention, health literacy, data systems quality, and health care management, with a reflection on persistent regional disparities in the health of the population, as well as health care availability, processes of demographic aging and related health indicators, and forecasts of health (64, 65).

These are inevitable for setting optimal health policies that will be in accordance with 17 SDGs. The main aim of the study is the analysis and evaluation of differences in 11 indicators of sustainable health and wellbeing in the EU countries.

The EU countries were analyzed based on eleven indicators that are part of the SDG 3. For analysis of the multidimensional view on health and wellbeing, the TOPSIS and ranking methods were chosen. The older EU Member States were better evaluated in terms of used criteria compared to the new, mostly post-communist countries. The best ranked countries in the first period were Sweden, Denmark, and the Netherlands using the TOPSIS method, while the best rated countries using the ranking method were Sweden, the Netherlands, and Ireland. In the first period, the worst positions were occupied by Lithuania, Romania, and Latvia regardless of the method used. In the second period, according to the TOPSIS method, the best countries, like in the first period, were Sweden, Denmark, and the Netherlands. No change for the three best countries in the second period was achieved using the ranking method. In the second period, Romania, Estonia, and Latvia were the three worst ranked countries using the TOPSIS method, while Lithuania, Romania, and Latvia were the worst rated countries according to the ranking method. The new EU countries had a lower HLY at birth compared to the older EU countries; they achieved higher SDRs due to tuberculosis, HIV, and hepatitis, higher standardized preventable and treatable mortality, and higher road traffic deaths indicator. The exposure to air pollution by particulate matter was higher in the new EU countries. All these factors negatively affected the rank of the new, mostly post-communist EU countries. The new EU countries therefore must more actively focus their efforts to improve the air and environment quality, which will result in a decline of the exposure to air pollution by particulate matter and can lead to a decline of death rates related to pollution. The worst rated countries must improve their strategies in health care system, which would then be able to more efficiently detect and treat preventable and treatable diseases. The higher road traffic deaths in the new, less developed EU countries could be related to poorer quality of roads, worse quality of vehicles used, and lack of highway networks; therefore, investments into road infrastructure are needed in these Member States. Naturally, each of us can contribute to good health and wellbeing by reducing obesity and smoking, and by improving our lifestyle (66, 67). These factors are also a part of the SDG 3. Prevention programs and systems of health literacy, which have to be a part of educational systems, play an important role in the process of reducing differences in health among countries (51, 68–70). In recent years, environmental literacy has been an important topic for many countries in relation to environmental strategies and reduction of environmental risks. All of these aspects represent challenges for further researches that would focus on multidimensional research of health systems' sustainability and sustainable health and wellbeing of entire population. The results of this study represent a valuable platform for creators of health and economic policies, and for creators of national health strategies and health plans. Additionally, the study results will support processes of methodological platform improvement for national and international comparative analyses and also for national and international benchmarking.

## Data Availability Statement

Publicly available datasets were analyzed in this study. This data can be found at: https://ec.europa.eu/info/strategy/international-strategies/sustainable-development-goals_en; https://ec.europa.eu/eurostat/web/main/data/database.

## Author Contributions

SM: conceptualization, methodology, formal analysis, investigation, data curation, writing—original draft preparation, visualization, writing—review, and editing. BG: conceptualization, investigation, resources, writing—original draft preparation, writing—review and editing, supervision, project administration, and funding acquisition. All authors read and approved the published version of the manuscript.

## Funding

This research was supported by the Scientific Grant Agency of the Ministry of Education, Science, Research, and Sport of the Slovak Republic and the Slovak Academy Sciences as part of the re-search project VEGA 1/0590/22: Exploration of natural, social and economic potential of areas with environmental burdens in the Slovak Republic for the development of specific forms of domestic tourism and quantification of environmental risks. This research was funded by the Slovak Research and Development Agency under the contract No. APVV-17-0360: Multidimensional analysis of significant determinants of public procurement efficiency with emphasis on the application of Health Technology Assessment in the procurement preparation phase.

## Conflict of Interest

The authors declare that the research was conducted in the absence of any commercial or financial relationships that could be construed as a potential conflict of interest.

## Publisher's Note

All claims expressed in this article are solely those of the authors and do not necessarily represent those of their affiliated organizations, or those of the publisher, the editors and the reviewers. Any product that may be evaluated in this article, or claim that may be made by its manufacturer, is not guaranteed or endorsed by the publisher.

## References

[1] GBD. Measuring the health-related Sustainable Development Goals in 188 countries: a baseline analysis from the Global Burden of Disease Study 2015. Lancet. (2016) 388:1813–50. 10.1016/S0140-6736(16)31467-2 27665228 PMC5055583

[2] Scrutton J Holley-Moore G Bamford SM. Creating a *Sustainable 21st Century Healthcare System*. The International Longevity Centre—UK (ILC-UK) (2015). Available online at: http://www.ilcuk.org.uk/ (accessed August 2, 2021).

[3] Hejduková P Kureková L. National health systems' performance: evaluation WHO indicators. Procedia Soc Behav Sci. (2016) 230:240–8. 10.1016/j.sbspro.2016.09.03130410738

[4] Pacáková V Kopecká L. Comparing inequalities in health outcomes in European countries. J Int Stud. (2018) 11:215–27. 10.14254/2071-8330.2018/11-4/1527558269

[5] Jakubowska A. Health and limitations in health as the determinant of human capital eff ectiveness: perspective of the EU Member States. J Int Stud. (2016) 9:240–51. 10.14254/2071-8330.2016/9-1/18

[6] Jakubowska A Bilan S Werbiński J. Chronic diseases and labour resources: “Old and new” European Union member states. J Int Stud. (2021) 14:129–38. 10.14254/2071-8330.2021/14-1/9

[7] Ivaldi E Testi A. Socio-economic conditions and health in Europe: a comparison among the 27 EU countries. In: Rosen JD, Eliot AP, editors. Social Inequalities. New York, NY: Nova Science Publishers (2011). p. 127–50.

[8] Lyeonov S Bilan S Yarovenko H Ostasz G Kolotilina O. Country's health profile: social, economic, behavioral and healthcare determinants. Econ Sociol. (2021) 14:322–40. 10.14254/2071-789X.2021/14-3/1723239202

[9] Pérez C Fernández C Méndez V Méndez P Fernández A. Evolution of GDP and its impact on the pharmaceutical sector of Ecuador (2007-2016). J Int Stud. (2018) 11:288–96. 10.14254/2071-8330.2018/11-1/22

[10] Paul P Hakobyan M Valtonen H. The association between self-perceived health status and satisfaction with healthcare services: evidence from Armenia. BMC Health Serv Res. (2016) 16:67. 10.1186/s12913-016-1309-6 26892950 PMC4759944

[11] Hunter DJ Kieslich K Littlejohns P Staniszewska S Tumilty E Weale A. Public involvement in health priority setting: future challenges for policy, research and society. J Health Organ Manag. (2016) 30:796–808. 10.1108/JHOM-04-2016-0057 27468775

[12] Romanelli M. New technologies for sustainable health care. In: Borangiu T, Dragoicea M, Nóvoa H, editors. Exploring Services Science. IESS 2016. Lecture Notes in Business Information Processing. Cham: Springer (2016). p. 247.

[13] Brӑtucu G Tudor AIM Dovleac L Sumedrea S Chiuţ IB Trifan A. The impact of new technologies on individuals' health perceptions in the European Union. Sustainability. (2020) 12:10349. 10.3390/su122410349

[14] Sun D Ahn H Lievens T Zeng W. Evaluation of the performance of national health systems in 2004-2011: an analysis of 173 countries. PLoS ONE. (2017) 12:e0173346. 10.1371/journal.pone.0173346 28282397 PMC5345793

[15] Bem A Predkiewicz K Predkiewicz P Ucieklak-Jez P. Determinants of hospital's financial liquidity. Proc Econ Finance. (2014) 12:27–36. 10.1016/S2212-5671(14)00317-7

[16] Sopko J Kočišová K. Key indicators and determinants in the context of the financial aspects of health systems in selected countries. Adiktologie. (2019) 19:189–202. 10.35198/01-2019-004-0003

[17] Briestenský R Ključnikov A. Identification of the key factors for successful hospital management in Slovakia. Adiktologie. (2019) 19:203–11. 10.35198/01-2019-004-0004

[18] Grasso M Canova L. An assessment of the quality of life in the European Union based on the social indicators approach. Soc Indic Res. (2008) 87:1–25. 10.1007/s11205-007-9158-7

[19] Rogge N Nijverseel I. Quality of life in the European Union: a multidimensional analysis. Soc Indic Res. (2019) 141:765–89. 10.1007/s11205-018-1854-y

[20] Aillon JL Dal SE. Health and Degrowth', a New Paradigm in the Field of Sustainability. Leipzig: Paper presented at the Fourth International Conference on Degrowth for Ecological Sustainability and Social Equity (2014).

[21] Borowy I. Economic growth and health: evidence, uncertainties and connections over time and place. In: Borowy I, Schmelzer M, editors. History of the Future of Economic Growth. Milton Park: Routledge (2017). p. 129–53.

[22] Missoni E. Degrowth and health: local action should be linked to global policies and governance for health. Sustain Sci. (2015) 10:439–50. 10.1007/s11625-015-0300-1

[23] Borowy I Aillon JL. Sustainable health and degrowth: Health, health care and society beyond the growth paradigm. Soc Theory Health. (2017) 15:346–68. 10.1057/s41285-017-0032-7

[24] Eurostat. Sustainable Development in the European Union: Monitoring Report on Progress Towards the SDGs in an EU Context – 2021 edition. Luxembourg: Publications Office of the European Union (2021). p. 407.

[25] Eurostat. Sustainable Development in the European Union: A Statistical Glance from the Viewpoint of the UN Sustainable Development Goals – 2016 edition. Luxembourg: Publications Office of the European Union (2016). p. 163.

[26] European, Comission,. Sustainable Development Goals. Available online at: https://ec.europa.eu/info/strategy/international-strategies/sustainable-development-goals_en (accessed September 23, 2021).

[27] United, Nations,. The 17 Goals | Sustainable Development. Available online at: https://sdgs.un.org/goals (accessed September 22, 2021).

[28] Megyesiova S Lieskovska V. Analysis of the sustainable development indicators in the OECD countries. Sustainability. (2018) 10:4554. 10.3390/su1012455430632043

[29] Szymańska A. Reducing socioeconomic inequalities in the European Union in the context of the 2030 agenda for sustainable development. Sustainability. (2021) 13:7409. 10.3390/su13137409

[30] Eurostat. Database. Available online at: https://ec.europa.eu/eurostat/web/main/data/database (accessed September 21, 2021).

[31] OECD & Joint Research Centre. Handbook on Constructing Composite Indicators: Methodology and User Guide. Paris: OECD & Joint Research Centre, OECD (2008).

[32] Kroll TC Wölfl S. Ranking: a closer look on globalisation methods for normalisation of gene expression arrays. Nucleic Acids Res. (2002) 30:e50. 10.1093/nar/30.11.e50 12034851 PMC117212

[33] Jencks SF Huff ED Cuerdon T. Change in the quality of care delivered to medicare beneficiaries, 1998-1999 to 2000-2001. JAMA. (2003) 289:305–12. 10.1001/jama.289.3.305 12525231

[34] Roszkowska E. Multi-criteria decision making models by applying the TOPSIS method to crisp and interval data. Multiple Crit Decis Mak. (2011) 6:200-30.

[35] Pawlik A Dziekanski P Przybytniowski JW. Influence of financial variables on the development of rural communes of Eastern Poland in 2009–2018. Risks. (2021) 9:145. 10.3390/risks9080145

[36] Cheba K Bak I. Environmental production efficiency in the European Union countries as a tool for the implementation of goal 7 of the 2030 agenda. Energies. (2021) 14:4593. 10.3390/en14154593

[37] Zhang Z Mao C Shi Z Kou X. The amino acid metabolic and carbohydrate metabolic pathway play important roles during salt-stress response in tomato. Front Plant Sci. (2017) 8:1231. 10.3389/fpls.2017.01231 28769946 PMC5511834

[38] Vassoney E Mochet AM Desiderio E Negro G Pilloni MG Comoglio C. Comparing multi-criteria decision-making methods for the assessment of flow release scenarios from small hydropower plants in the alpine area. Front Env Sci. (2021) 9:635100. 10.3389/fenvs.2021.635100

[39] Li ZG Wei HA. Comprehensive evaluation of China's TCM medical service system: an empirical research by integrated factor analysis and TOPSIS. Front Public Health. (2020) 8:532420. 10.3389/fpubh.2020.532420 33117767 PMC7550738

[40] Srikrishna S Sreenivasulu RA Vani S. A new car selection in the market using TOPSIS technique. Int J Eng Res Gen Sci. (2014) 4:177-81.

[41] Hauke J Kossowski T. Comparison of values of Pearson's and Spearman's Correlation coefficients on the same sets of data. Quaestiones Geographicae. (2011) 30:87-93. 10.2478/v10117-011-0021-1

[42] Statistics How To,. Spearman Rank Correlation. (2021). Available online at: https://www.statisticshowto.com/probability-and-statistics/correlation-coefficient-formula/spearman-rank-correlation-definition-calculate/ (accessed September 25, 2021).

[43] Song HY Park S. An analysis of correlation between personality and visiting place using Spearman's rank correlation coefficient. KSII Transac Internet Inform Syst. (2020) 14:005. 10.3837/tiis.2020.05.005

[44] Simionescu M Pelinescu E Khouri S Bilan S. The main drivers of competitiveness in the EU-28 countries. J Competitive. (2021) 13:129–45. 10.7441/joc.2021.01.08

[45] Boikova T Zeverte-Rivza S Rivza P Rivza B. The determinants and effects of competitiveness: the role of digitalization in the European economies. Sustainability. (2021) 13:11689. 10.3390/su132111689

[46] European Health Interview Survey (EHIS). Available online at: https://ec.europa.eu/eurostat/cache/metadata/en/hlth_det_esms.htm (accessed September 26, 2021).

[47] OECD. Health Systems Characteristics Survey. (2016). Available online at: https://qdd.oecd.org/subject.aspx?Subject=hsc (accessed October 18, 2021).

[48] Rahman MM Khanam R Rahman M. Health care expenditure and health outcome nexus: new evidence from the SAARC-ASEAN region. Global Health. (2018) 14:113. 10.1186/s12992-018-0430-1 30466452 PMC6249744

[49] Karanikolos M Mackenbach JP Nolte E Stuckler D McKee M. Amenable mortality in the EU-has the crisis changed its course? Eur J Public Health. (2018) 28:864–9. 10.1093/eurpub/cky116 29982338

[50] Dieleman JL Campbell M Chapin A Eldrenkamp E Fan VY Haakenstad A. Future and potential spending on health 2015–40: development assistance for health, and government, prepaid private, and out-of-pocket health spending in 184 countries. Lancet. (2017) 389:2005–30. 10.1016/S0140-6736(17)30873-5 28433260 PMC5440765

[51] Gavurova B Kocisova K Sopko J. Health system efficiency in OECD countries: dynamic network DEA approach. Health Econ Rev. (2021) 11:40. 10.1186/s13561-021-00337-9 34642864 PMC8513208

[52] Cylus J Papanicolas I Smith PC. Using data envelopment analysis to address the challenges of comparing health system efficiency. Global Policy. (2017) 8:60–8. 10.1111/1758-5899.12212

[53] Nolasco A Pereyra-Zamora P Sanchis-Matea E Tamayo-Fonseca N Caballero P Melchor I. Economic crisis and amenable mortality in Spain. Int J Environ Res Public Health. (2018) 15:2298. 10.3390/ijerph15102298 30347682 PMC6211017

[54] Ranabhat CL Atkinson J Park MB Kim CB Jakovljevic M. The influence of universal health coverage on life expectancy at birth (LEAB) and healthy life expectancy (HALE): a multi-country cross-sectional study. Front Pharmacol. (2018) 9:960. 10.3389/fphar.2018.00960 30279657 PMC6153391

[55] Kohl S Schoenfelder J Fügener A Brunner JO. The use of data envelopment analysis (DEA) in healthcare with a focus on hospitals. Health Care Manage Sci. (2018) 23:1–43. 10.1007/s10729-018-9443-9 29600469

[56] Mihalache IC. Health state of human capital in the economic theory. Postmodern Open. (2019) 10:182–92. 10.18662/po/102

[57] Novignon J Olakojo SA Nonvignon J. The effects of public and private health care expenditure on health status in sub-Saharan Africa: new evidence from panel data analysis. Health Econ Rev. (2012) 2:22. 10.1186/2191-1991-2-22 23232089 PMC3533939

[58] Kozlova OA Nifantova RV Makarova MN. Methods of the assessment of economic losses caused by the mortality of the population employed in regional economy. Econ Reg. (2017) 13:511–23. 10.17059/2017-2-16

[59] Ozcan YA Khushalani J. Assessing efficiency of public health and medical care provision in OECD countries after a decade of reform. CEJOR. (2017) 25:325–43. 10.1007/s10100-016-0440-0

[60] Rigelsky M Ivankova V Gavurova B Mudrik M. The effect of the minimum wage on smoking-related indicators in selected OECD countries. Equil Quarter J Econ Econ Policy. (2020) 15:439–61. 10.24136/eq.2020.020

[61] Skvarciany V Lapinskaite I Volskyte G. Circular economy as assistance for sustainable development in OECD countries. Oeconomia Copernicana. (2021) 12:11–34. 10.24136/oc.2021.001

[62] World Health Organization. World Health Statistics 2018: Monitoring Health for the SDGs Sustainable Development Goals. World Health Organization (2018). Available online at: https://apps.who.int/iris/handle/10665/272596 (accessed February 9, 2020).

[63] Svabova L Tesarova EN Durica M Strakova L. Evaluation of the impacts of the COVID-19 pandemic on the development of the unemployment rate in Slovakia: counterfactual before-after comparison. Equil Quarter J Econ Econ Policy. (2021) 16:261–84. 10.24136/eq.2021.010

[64] Liu N Xu Z Skare M. The research on COVID-19 and economy from 2019 to 2020: analysis from the perspective of bibliometrics. Oeconomia Copernicana. (2021) 12:217–68. 10.24136/oc.2021.009

[65] Małkowska A Urbaniec M Kosała M. The impact of digital transformation on European countries: insights from a comparative analysis. Equil Quarter J Econ Econ Policy. (2021) 16:325–55. 10.24136/eq.2021.012

[66] Rolova G Gavurova B Petruzelka B. Exploring health literacy in individuals with alcohol addiction: a mixed methods clinical study. Int J Env Res Public Health. (2020) 17:6728. 10.3390/ijerph17186728 32942763 PMC7558488

[67] Zborník TS Miovský M. Comprehensive evaluation of the online life-long education course prevention and treatment of substance use disorders for physicians in the Czech Republic: study protocol. Adiktologie. (2021) 21:179–83. 10.35198/01-2021-003-0004

[68] Barros R Serrano PI Tufró F Canay R Carroli M Felix TR. Client's characterization in the Community Treatment approach: methodological foundations and evidence. Adiktologie. (2021) 21:85–94. 10.35198/01-2021-002-0003

[69] Gavurova B Rigelsky M Ivankova V. Perceived health status and economic growth in terms of gender-oriented inequalities in the OECD countries. Econ Sociol. (2020) 13:245-57. 10.14254/2071-789X.2020/13-2/16

[70] Pipová H Dostál D Dolejš M Kafetsios K Suchá J. Validation of the Czech modified Yale food addiction scale in a representative sample of adolescents: connections with body mass index and impulsivity. Adiktologie. (2021) 21:105–14. 10.35198/01-2021-002-0006

